# Effects of corticosteroids after total reapair of congenital heart desease with extracorporeal circulation

**DOI:** 10.1186/1749-8090-8-S1-O6

**Published:** 2013-09-11

**Authors:** V Ivancan, Z Colak, S Konosic, R Gabelica, D Anic, D Belina, M Petricevic

**Affiliations:** 1 Department of Anesthesiology and Intensive Care, University Hospital Center Zagreb, Zagreb, Croatia; 2 Department of Cardiac Surgery, University Hospital Center Zagreb, Zagreb, Croatia

## Background

Nowadays, the use of cardiopulmonary bypass (CPB) followed by systemic hypothermia is common in cardiac surgery procedures. CPB causes a systemic inflammatory response syndrome (SIRS) that is markedly expressed in congenital caridiosurgical surgery programe resulting with deleterious consequences. Those effects are mediated through cytokines and others mediators of acute inflammatory response in circulation, which may lead to low cardiac output syndrome, multiorgan failure and lethal outcome after surgical total correction of congenital heart disease. The best method for SIRS prevention remains unclear, although some authors suggest perioperative use of corticosteroids. The study sough to evaluate the impact of perioperative corticosteroids on SIRS extent as well as clinical outcomes following total correction of congenital heart disease.

## Methods

The study was conducted as prospective randomized controlled trial. We enrolled 60 patients who were scheduled for total correction of congenital heart disease.

In this study we examine the effects of corticosteroids in different doses and different time. Patients were divided into three groups with respect to perioperative corticosteroids administration regime.

## Results

More favorable clinical outcomes as well as laboratory findings were obtained in group of patients receiving high doses of corticosteroids. The best clinical outcome was observed in group receiving (30mg/kg metilprednisolon after induction in anesthesia), where we found the shortest ICU stay (2,6 days vs. 7,9 – Ib; 4,8 – II; 5,4 –IIIa; 8,7 – IIIb), the lowest requirements for inotropic support (40 % vs. 80 % - Ib; 75 % - II; 100 % - IIIa, IIIb), the shortest ventilation time (0,7 hours vs. 24,6 – Ib; 9,75 – II; 9,70 – IIIa; 16,6 – IIIb).

## Conclusion

Our study presumably described that perioperative corticosteroids provide more favorable outcomes in group of patients who undergo total correction of congenital disease. However, further prospective multicentric randomized trials are required with aim to elucidate optimal timing and dosing regimens of perioperative corticosteroids.

